# A systematic review on Transoral robotic surgery (TORS) for carcinoma of unknown primary origin: Has tongue base mucosectomy become indispensable?

**DOI:** 10.1111/coa.13565

**Published:** 2020-05-25

**Authors:** Stijn van Weert, Johannes A. Rijken, Francesca Plantone, Elisabeth Bloemena, Marije R. Vergeer, Birgit I. Lissenberg‐ Witte, C. René Leemans

**Affiliations:** ^1^ Department of Otolaryngology/Head and Neck Surgery Amsterdam UMC Vrije Universiteit Amsterdam Amsterdam The Netherlands; ^2^ Department of Otorhinolaryngology University of Bari Bari Italy; ^3^ Department of Pathology and Department of Oral and Maxillofacial Surgery/Pathology Amsterdam UMC and Academic Centre for Dentistry Amsterdam (ACTA) Cancer Center Amsterdam Vrije Universiteit Amsterdam Amsterdam The Netherlands; ^4^ Department of Radiation Oncology Amsterdam UMC Vrije Universiteit Amsterdam Amsterdam The Netherlands; ^5^ Department of Epidemiology and Biostatistics Amsterdam UMC Vrije Universiteit Amsterdam Amsterdam The Netherlands

**Keywords:** cancer, head and neck surgery, neck lump

## Abstract

**Background:**

Transoral robotic surgery (TORS) is increasingly used in head and neck surgery and in carcinoma of unknown primary (CUP) origin specifically. Due to the rising incidence of human papillomavirus (HPV)‐related oropharyngeal squamous cell carcinoma (OPSCC), there is a rationale for finding ways to de‐escalate treatment strategies. This review aims to test the hypothesis that TORS is a meaningful adjunct in the diagnostic (and therapeutic) pathway in CUP in head and neck.

**Methods:**

A structured search of the literature was performed with the search terms ‘TORS’ and ‘Carcinoma of Unknown Primary’.

**Results:**

Two hundred and seventy four cases of CUP in which TORS was used were identified for further analysis. Workup for CUP was comparable in all series with regard to physical examination, fine and/or gross needle examination of cervical nodes, fibre optic endoscopy, imaging and robot assisted mucosectomy of the base of tongue (BOT). Identification rate of the primary tumour was 72% on average (range 17%‐ 90%), and 55%‐ 96% were HPV positive. Clear margins were achieved in 60% (range 0%‐85%) of resected occult tumours. Complication rate of TORS BOT mucosectomy was low with mainly grade I‐III sequelae according to Clavien–Dindo.

**Conclusions:**

Transoral robotic surgery seems to be a useful and safe adjunct in the diagnostic and therapeutic pathway in case of CUP in an era of increasing incidence of HPV‐positive OPSCC.

**Key points**

- Transoral robotic surgery (TORS) is emerging in the field of diagnostics and treatment of occult head and neck tumours
- This systematic review sets out to analyse earlier reported results and find answers to whether TORS is a true useful adjunct in the work around in cervical unknown primary (CUP)
- TORS seems to be beneficial with a higher identification rate of occult primaries compared to classic endoscopy with biopsies
- TORS in CUP may lead to de‐intensification of treatment in oropharyngeal squamous cell carcinoma, mainly in case of HPV positivity.
- TORS is a meaningful adjunct in the diagnostic and therapeutic pathway in CUP and should be considered to be structurally implemented in the guidelines of CUP.

## 1 INTRODUCTION

Carcinoma of unknown primary (CUP) metastatic to cervical lymph nodes represents 1%‐5% of all cases of head and neck malignancies, and consists mainly of squamous cell carcinoma (SCC) (50%‐70%).^1^, ^2^ Once identified, the majority of initially occult oropharyngeal cancers turn out to be high‐risk human papillomavirus (hrHPV) related. As a result of the increase in HPV‐related oropharyngeal squamous cell carcinoma (OPSCC), the incidence of CUP is increasing.^3^, ^4^ The reported HPV prevalence for CUP in head and neck ranges between 22%‐91% worldwide. p16 protein expression is considered to be a good surrogate marker of HPV‐status (although 15% of p16 positive tumours is HPV DNA negative) which is currently the most important independent prognostic factor in OPSCC.^5^ There is no general consensus on preferred diagnostic investigations in CUP, although physical examination including (office‐based) endoscopy and diagnostic imaging such as computed tomography (CT), magnetic resonance (MR) imaging and positron‐emission tomography (PET) is routinely performed in most institutions. The improvement of imaging techniques, high‐definition endoscopic instruments and the introduction of narrow‐band imaging (NBI) has significantly improved detection rates of the primary head and neck tumour over the years.^6^ Despite these efforts, approximately 50% of primary tumours remain undetected.^7^, ^8^ Identification of the primary tumour site is important for optimal treatment. The current standard treatment for (occult) OPSCC is based on either surgery and/or radiotherapy, both associated with comparable, high tumour control rates but with different side effects profiles and technical constraints.

In order to decrease the potential morbidity of open surgery, transoral approaches have been developed within the last decades, including transoral robotic surgery (TORS).

There is no general consensus in the various national guidelines such as the United States National Comprehensive Cancer Network (NCCN) and the British National Institute for Health and Care Excellence (NICE) on the role of TORS in CUP diagnostics.^7^, ^8^, ^9^, ^10^

The present study aims to determine the true benefit of TORS in detecting unknown primary tumours by conducting a systemic review of the literature.

## 2 METHODS

### 2.1 Ethical considerations

Institutional ethical approval needed not to be obtained for this systematic review for which publicly accessible data were used. None of the used data are individually traceable.

A MEDLINE (PubMed) search was performed with the search terms ‘TORS’ AND ‘Carcinoma of Unknown Primary’ using a combination of MeSH headings and keywords. The study was not designed to identify all studies on TORS in head and neck cancer, but to analyse those that focused on TORS for CUP. The search was limited to humans, clinical trials, randomised controlled trials (RCTs), case reports and English language articles from January 2013 to September 2018. Full articles of all citations resulting from this search were obtained. We scrutinised all articles for details of the methodology used to obtain an unknown primary tumour. Two reviewers independently screened all identified studies by title and abstract for further full‐text review and then independently reviewed these studies for eligibility. When multiple studies were published by a single institution, only the most recent study was included to avoid inclusion of the same patients more than once in this review. Disagreements were resolved by consensus. Data from the included studies were extracted and entered onto a Excel spreadsheet for collation and analysis.

## 3 RESULTS

A total of 274 cases of CUP were included from 12 case series (2013‐2018).^11^, ^12^, ^13^, ^14^, ^15^, ^16^, ^17^, ^18^, ^19^, ^20^, ^21^, ^22^ The preoperative workup was generally similar in all studies consisting of physical examination with flexible fibre optic laryngoscopy in all cases, imaging (CT and PET/CT in the vast majority, with or without MR imaging), panendoscopy under general anaesthesia with or without bilateral palatine tonsillectomy. Diagnostic workup data are listed in Table 1.

**Table 1:** Diagnostic pre‐TORS workup data in CUP

Author Year	Cases	CT	MRI	PET/CT	EUA + biopsies
Abuzeid et al^11^ 2013	1	1	—	1	1
Blanco et al^12^ 2013	4	N/A	N/A	N/A	N/A
Metha et al^13^ 2013	10	10	—	10	10
Patel et al^14^ 2013	47	38	3	27	18
Durmus et al^15^ 2014	22	22	—	22	22
Byrd et al^16^ 2014	22	22	—	19	9
Channir et al^17^ 2015	13	13	—	13	13
Geltzeiler et al^18^ 2016	50	50	—	50	23
Krishnan et al^19^ 2016	7	7	—	7	3
Hatten et al^20^ 2017	60	44	14	59	N/A
Winter et al^21^ 2017	32	13	17	32	13
Sudoko et al^22^ 2018	6	6	—	6	N/A

Abbreviations: CT, computed tomography; EUA, endoscopy under general anaesthesia; MRI, magnetic resonance imaging; N/A, not applicable; PET, positron‐emission tomography.

The average identification rate of the primary oropharyngeal tumour using TORS was 72% (range 17%‐90%).

In 142 cases, the primary tumour was identified in the BOT. Fifty four (54) cases involved the palatine tonsil. Five studies report on mucosectomy of the base of tongue (BOT) only (without palatine tonsillectomy) in case of CUP.^14^, ^16^, ^18^, ^20^, ^21^

The studies reviewed reported a range of 55%‐96% positivity of HPV/p16 in CUP. In 60% (range 0%‐85%) of all detected CUP, negative margins were observed after TORS resection.(Table 2).

**Table 2:** Identification rates and histopathological data of previous reports on TORS for CUP

Author Year	Identification rate (%)	Negative surgical margins^a^ (%)	p16/HPV positive (%)	Base of tongue (n)	Palatine tonsils (n)
Abuzeid et al^11^ 2013	1/1 (100%)	0/1 (0%)	N/A	1	—
Blanco et al^12^ 2013	1/4 (25%)	N/A	N/A	—	1
Metha et al^13^ 2013	9/10 (90%)	1/9 (11%)	80%	9	N/A
Patel et al^14^ 2013	34/47 (72%)	29/34 (85%)	55%	21^c^	13
Durmus et al^15^ 2014	17/22 (77%)	13/17 (77%)	95%	4	13
Byrd et al^16^ 2014	19/22 (86%)	10/19 (53%)	91%	16	3
Channir et al^17^ 2015	7/13 (54%)	3/7 (43%)	69%	7	N/A
Geltzeiler et al^18^ 2016	37/50 (74%)	19/37 (51%)	96%	32	5
Krishnan et al^19^ 2016	5/7 (71%)	3/5 (60%)	86%	5	N/A
Hatten et al^20^ 2017	48/60 (80%)	40/48 (83%)	92%	30^d^	18
Winter et al^21^ 2017	17/32 (53%)	N/A	72%	17^b^	N/A
Sudoko et al^22^ 2018	1/6 (17%)	1/1 (100%)	83%	1	N/A
Total	196/274 (72%)	119/196 (61%)			

Abbreviations: N, number of cases; N/A, not applicable.

^a^ Following the principles of most TORS protocols, margins above 2 mm were considered free.

^b^ In 2/17 cases, the primary was found in the contralateral palatine tonsil.

^c^ One case with involvement of tonsil and BOT registered as BOT.

^d^ Two cases involving the glossotonsillar sulcus.

The complications reported were re‐classified according to the Clavien–Dindo classification of surgical complications and are presented in Table 3.^11^, ^12^, ^13^, ^14^, ^15^, ^16^, ^17^, ^18^, ^19^, ^20^, ^21^, ^22^, ^23^

**Table 3:** Clavien–Dindo classification

Grade	Definition	Complication	Incidence
Grade I	Any deviation from normal postoperative course without intervention^a^	Pain	0.7%
Grade II	Pharmacological treatment required incl. blood transfusion/parenteral feeding	Peri‐operative feeding tube dependence	2.9%
Grade III	Requiring intervention (surgical, endoscopic, radiological)		
IIIa	Without general anaesthesia	NA	NA
IIIb	Requiring general anaesthesia	Bleeding	4.4%
Grade IV	Life‐threatening complication requiring ICU management	NA	0%
IVa	Single organ dysfunction	—	—
IVb	Multiorgan dysfunction	—	—
Grade V	Death of a patient	Death	0.4%

Note

Wound infections opened at the bedside.

^a^ Allowed: antiemetics, antipyretics, analgetics, diuretics, electrolytes and physiotherapy.

## 4 DISCUSSION

In the quest for improvement of identification rates of occult head and neck tumours TORS has been emerging over the recent years as a possible means of achieving this goal, subsequently leading to possible de‐intensification of treatment. The incidence of CUP is increasing due to the increase in HPV‐positive OPSCC. These HPV–related OPSCC's are associated with low primary tumour burden with an improved disease‐specific survival and overall survival compared to non‐HPV‐related OPSCC's. Since occult tumours often prove to be located in the oropharynx, a relatively large percentage of HPV‐positive OPSCC's present a CUP.^24^

In the non‐surgical workup, detection methods for CUP in the head and neck region changed significantly over the years, with the introduction of CT, MR, PET, NBI and, more recently, p16 and HPV DNA testing.^6^, ^24^

(18)F‐FDG‐PET/CT was used in the majority of cases reviewed. It should however be noted that the sensitivity and specificity for BOT lesions on (18)F‐FDG‐PET/CT is moderate due to physiological isotope uptake in the lymphoid tissue of the lingual tonsils, possibly leading to false‐positive results.^25^ Pattani et al^26^ reported that in their series of CUP all positive results in (18)F‐FDG‐PET/CT were confirmed during panendoscopy. In case of a negative PET/CT however, only in 10% of cases a primary tumour was identified during classic panendoscopy.

Magnetic resonance imaging is superior to CT in identifying the primary lesion. With the growing experience in using MR imaging with diffusion‐weighted imaging (DWI), there seems to be a place in the diagnostic workup for CUP. Noij et al recently described a high sensitivity for both DWI–MR (81.3%) and (18)F‐FDG‐PET/CT (93.8%) in detection of the primary lesion.^27^

With regard to imaging, there is a clear discrepancy in modalities used in prior studies. Winter et al used both MR (53%) and CT (47%) without clear argumentation. Hatten et al (29%) and Patel et al (6%) only used MR in selected cases.^14^, ^20^, ^21^ DWI is not mentioned in any of these reports.

**Figure 1:**
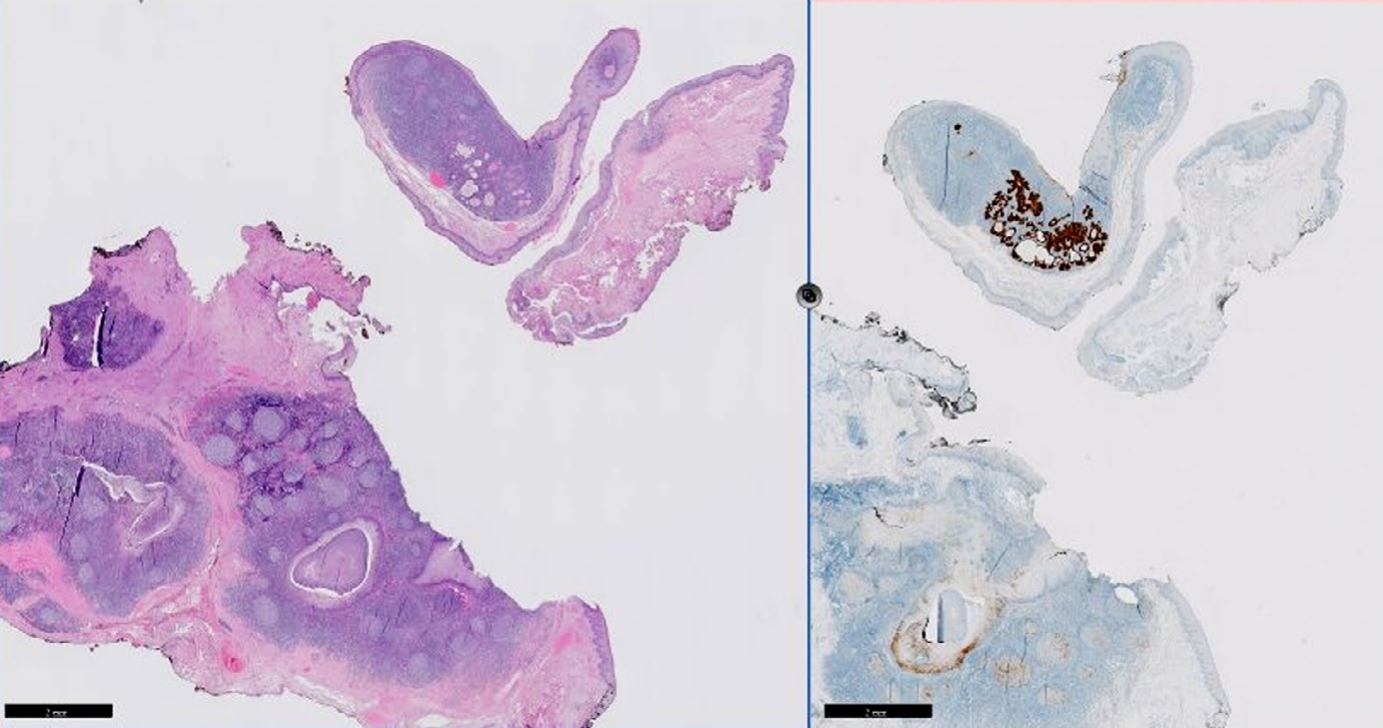
Small (2.2 mm) partly cystic squamous cell carcinoma detected in the crypts of the lymphoid tissue in the base of tongue in a patient with multiple unilateral metastases in the neck (left HE, right p16 immunostaining)

In the event that physical examination and imaging fail to identify the primary lesion or to target the area suspected for a primary lesion, TORS can be considered in the diagnostic workup for CUP. By performing a diagnostic oropharyngeal resection, there is a reasonable chance of achieving clear margins in small occult disease that may often be detectable by immunostaining (ie p16) only; Figure 1 shows an example of a small primary with positive p16 immunostaining. Different cut‐off dimensions for adequate surgical margins are used in literature varying from 2‐5 mm for a clear margin where others do not define a clear margin.^14^, ^24^, ^28^

This review shows a superior detection rate for occult tumours by TORS: 72% vs. 41% compared to conventional EUA with biopsies, respectively.^29^, ^30^, ^31^

With respect to techniques used for TBM, TORS has been preceded by transoral laser microsurgery (TLM) and endoscopic electrocautery assisted TBM has recently been touched upon by Davies‐Husband.^32^ This technique—reportedly easily acquired with experience in tonsillectomy and TLM—might be a cost‐effective alternative when the laser and/ or robotic system are not available. The identification rate of occult BOT primaries in this small series (n = 9) was 44.4 per cent. One clear disadvantage of TLM vs TORSis the limited line of sight and percentage of proper exposition which make TORS applicable in a relatively larger population.^21^ Recent reports have critically looked at the identifications rates using different techniques. Farooq et al found a pooled (TLM and TORS) identification rate of 78% (both tonsil and BOT) with 91% for TLM. An important footnote concerning these numbers is the relatively small number of studies on CUP and TLM (n = 3).^33^

One of the reasons of the robotic's system superior detection rate of mainly occult BOT tumours is the camera of the Da Vinci^®^ robot (Intuitive Surgical) which permits a high‐definition three‐dimensional magnified view of the oropharynx for optimal visualisation of surface mucosa and allows for a proper mucosectomy of the BOT. A recent systematic review by Fu et al^34^ on identifying the unknown primary (robot‐assisted and non‐robotic transoral surgery) has reported on eight studies (n = 139) from North American institutions reporting similar diagnostic rates of approximately 80%.

For the current review, recently published studies conducted in the UK, Australia and Denmark each using their national guidelines with differences in terms of diagnostic workup and patient selection were considered.

The results of the current study could be influenced by confounding factors such as the differences in diagnostic workup as reported in Table 2.

**Figure 2:**
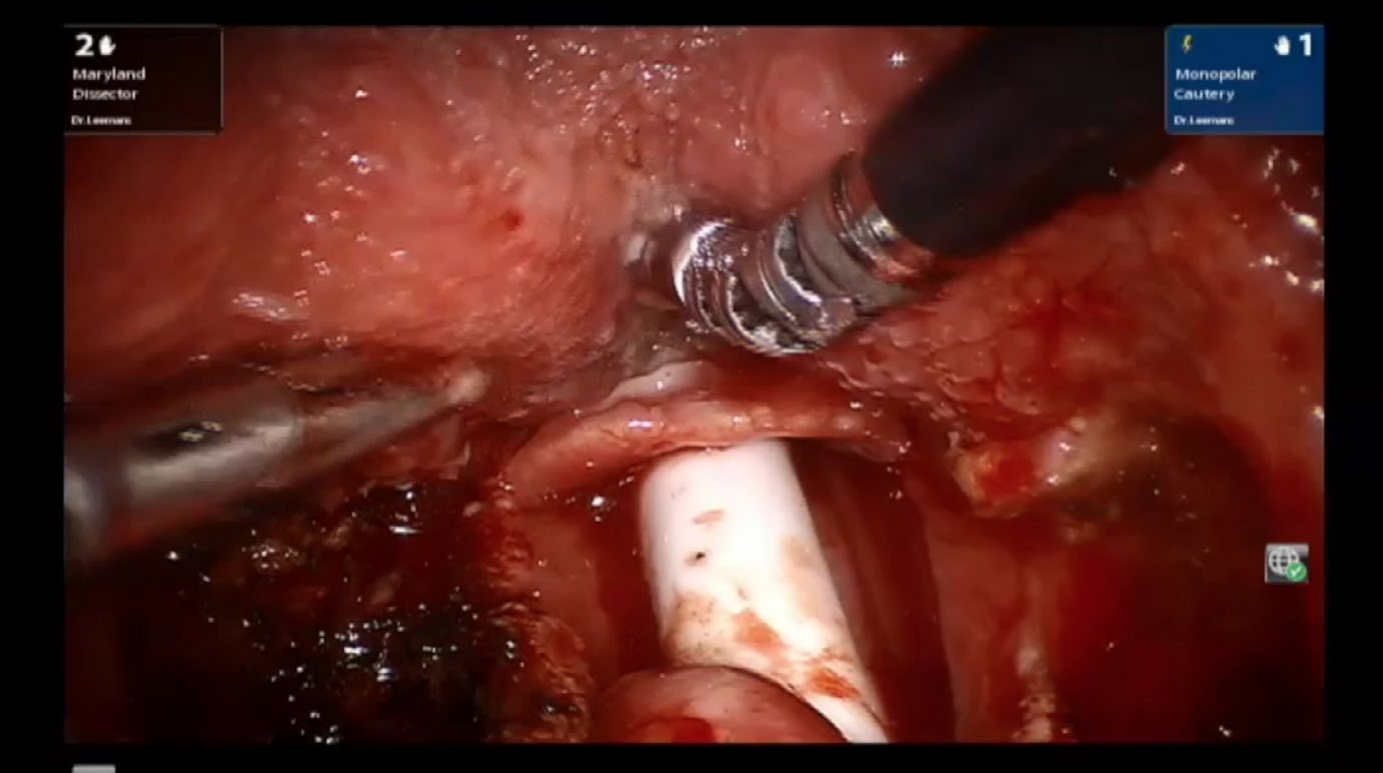
A and B, Left sided base of tongue mucosectomy with the Da Vinci Si. Note the forceps on the left and the monopolar spatula on the right. The procedure is commenced by a midline incision in the base of tongue

The emphasis in most reports on TORS for CUP is on the BOT based on the fact that an occult primary of the palatine tonsil would be identified by classic dissection tonsillectomy as well. Tonsillectomy combined with panendoscopy is not routinely performed in all reports analysed. Palatine tonsillectomy has shown to provide cancer detection rates superior to biopsy of tonsillar tissue.^35^ Differences in the surgical technique—described in five papers—of BOT mucosectomy might influence results in terms of identification rates.^11^, ^13^, ^17^, ^19^, ^21^ Paleri et al^36^ recommend to use a midline incision for two separate BOT specimens for proper orientation and reduction of specimen trauma (see Figure 2A,B which show a left sided BOT mucosectomy). Pathology laboratory protocols differ widely, potentially leading to large differences in identification rate in different centres. Ideally, step serial sectioning (SSS) should be employed for the entire specimen supplied. This technique is however time‐consuming, expensive and no standard of care yet as it is for sentinel lymph node analysis.^21^ Uniform algorithms are proposed in recent literature.^21^, ^37^

Severe complications (Clavien–Dindo grade III‐V) after TORS are relatively rare. Postoperative bleeding represented the most frequent sequelae and only needed intervention (Clavien–Dindo grade III) in selected cases.^23^, ^38^, ^39^ FU et al report an overall 7% complication rate (n = 139) with postoperative bleeding as the most common complication for TORS and TLM combined.^34^ In the current TORS‐only study (n = 274), a slightly higher complication rate of 11% was found.

The actual benefit of TORS for CUP is possible de‐intensification of treatment as a consequence of identifying (and/or resecting) the primary site. The incentive for decreasing toxicity in this group of relatively young patients is clear and is primarily focused on reduction of long‐term dysphagia, avoiding carotid atherosclerosis and xerostomia. A successful detection/resection of the primary tumour is essential in reducing long‐term side effects associated with pharyngeal radiation such as xerostomia and dysphagia.^20^ As described by Graboyes et al,^40^ a proper algorithm for CUP in p16 + cases specifically can lead to avoiding this toxicity by eliminating the primary site from the RT field when the primary was detected and removed with adequate margins or in case of undetected primary with complete ipsilateral palatine and lingual tonsillectomy including normal endoscopy findings.

Patel et al describe a similar post‐TORS RT regimen with a currently observed inter‐institutional variability.^29^

Current de‐escalation trials such as PATHOS^41^ and ECOG E‐3311^42^ are looking at the possibility of de‐intensifying adjuvant treatment for HPV‐positive OPSCC's after transoral surgery. The study design of PATHOS (recruiting at present) has three arms for three risk groups; one without (conventional) adjuvant treatment (low risk), one with radiation dose de‐escalation (medium risk) and one with radiotherapy only as opposed to conventional combined modality treatment with cisplatin (high risk). For the ECOG E‐3311, these are four arms; arm A only transoral surgery (TOS), arm B TOS and low dose IMRT, arm C TOS and standard‐dose IMRT and arm D TOS and concurrent chemoradiation. Endpoints of these trials are notably QoL and swallowing performance measured with the MD Anderson Dysphagia Inventory (MDADI) after 1‐2 years.

Ma et al recently presented their results (phase II MC1273 study; 2‐year follow‐up) for dose de‐escalation in adjuvant chemoradiation (weekly docetaxel combined with 30‐ 36 Gray (Gy) twice daily over a two week period) in HPV‐positive OPSCC's. In case of extracapsular extension (ECE), a simultaneous boost was given to a total dose of 36 Gy. Their primary results look promising with a good loco‐regional control (95%) and disease‐free survival (89%). Moreover, side effects were reduced, confirmed by zero tube dependence and relatively low toxicity.^43^ Longer follow‐up and randomised trials such as PATHOS and ECOG E‐3311 are needed to confirm these findings. The results of both PATHOS and ECOG E‐3311 (expected between 2021‐2023) will undoubtedly point out whether de‐intensification of adjuvant treatment in HPV‐positive OPSCC is the right way.^41^, ^42^

In case of limited nodal involvement (ie one positive lymph node without ECE), selective neck dissection is sufficient for controlling cervical disease without the need for adjuvant treatment. In case of this limited nodal burden which in itself does not necessitate adjuvant treatment, additional BOT resection in case of positive or close margins after TBM might avoid multi‐modality treatment. In this respect, proper orientation of the initial specimen and good communication with the pathologist is key.

Finally, the costs for acquisition and maintenance of the da Vinci^®^ robot can be a threshold in implementing TORS in the work around in CUP of the head and neck. In 2014, Byrd et al reported on the cost‐effectiveness of TORS in case of CUP. They also advocated a sequential strategy of primary EUA with tonsillectomy followed by BOT mucosectomy when necessary in a second procedure. In their series, this seemed more cost‐effective due to shorter admission time due to less postoperative pain.^16^

## 5 CONCLUSIONS

This systematic review supports the added value of TORS for the identification of primary HNSCC of unknown origin in an era of increasing incidence of HPV‐positive OPSCC. The vast majority of primary (mainly HPV positive) tumours is found through TORS, and the complication rate is relatively low.

The BOT harbours the majority of occult tumours which is emphasised by the identifications rates of BOT mucosectomy.

Transoral robotic surgery for CUP may lead to de‐intensification of treatment by refraining from pharyngeal radiation and/or dose de‐escalation in select cases but results of forenamed de‐intensification trials need to be awaited. Prerequisites for TORS in CUP of the head and neck are well defined and uniform surgical and histopathological protocols.

## CONFLICT OF INTEREST

No conflict of interest to declare for all authors.

## Data Availability

The data that support the findings of this study are openly available https://www.ncbi.nlm.nih.gov/pubmed.
